# Novel loss-of-function mutations in VPS13A cause chorea-acanthocytosis in two families

**DOI:** 10.3389/fneur.2025.1643889

**Published:** 2025-08-22

**Authors:** Xiao-Hong Lin, Bin Xiao, Ru-Kai Chen, Jia-Ying Chen, Nai-Qing Cai, Chun-Yan Cao, Li-Qiong Zhan

**Affiliations:** ^1^Department of Rehabilitation Medicine, The First Affiliated Hospital, Fujian Medical University; National Regional Medical Center, Binhai Campus of the First Affiliated Hospital, Fujian Medical University, Fuzhou, China; ^2^Department of Neurology of First Affiliated Hospital, Fujian Medical University, Fuzhou, China; ^3^Department of Geriatrics, Fujian Medical University Union Hospital, Fuzhou, China; ^4^Department of Neurology, Henan Provincial People's Hospital, People's Hospital of Zhengzhou University, Zhengzhou, China

**Keywords:** chorea-acanthocytosis, gene mutations, VPS13A, neurology, whole-exome sequencing

## Abstract

**Introduction:**

Chorea-acanthocytosis (ChAc) is the most common subtype of neuroacanthocytosis (NA) caused by mutations in VPS13A (vacuole protein sorting-associated protein 13A). ChAc is characterized by the presence of spherocytes and neurological symptoms. This article reports two families with ChAc and summarizes some suggestive characteristics, providing an effective basis for clinicians to screen ChAc in the early stage and effectively reduce the misdiagnosis and missed diagnosis of this disease.

**Methods:**

We first performed whole-exome sequencing (WES) and confirmed three NA cases in two families. Detailed clinical and peripheral blood smear analyses are presented, supplemented by molecular electron microscopy to assess erythrocyte ultrastructure. To further evaluate the functional impact of candidate variants, we additionally performed RNA splicing analysis.

**Results:**

Three ChAc cases in two families were identified. Clinically, almost all cases presented initial movement disorders, and Elevated creatine kinase (CK) level. Besides, both peripheral blood smear and scanning electron microscopy revealed characteristic acanthocytes.

**Conclusions:**

This study provides key clinical indicators for early ChAc screening: early movement disorders combined with persistently elevated CK levels and significant acanthocytosis on peripheral blood smear. We further identified three novel VPS13A mutations, expanding the variant spectrum and confirming clinical heterogeneity in ChAc.

## 1 Introduction

Neuroacanthocytosis syndromes encompass a group of rare, genetically heterogeneous neurological disorders defined by the presence of acanthocytes (abnormally spiked erythrocytes) in peripheral blood and progressive neurodegeneration (1) encompassing principal subtypes such as autosomal recessive chorea-acanthocytosis (ChAc) and X-linked McLeod syndrome (MLS). These conditions predominantly affect the basal ganglia and manifest with a spectrum of movement disorders, including chorea, dystonia, and Parkinsonism, alongside neuropsychiatric symptoms such as cognitive decline and behavioral changes (2). NA syndromes arise from mutations affecting membrane-associated proteins or lipid metabolism, leading to both erythrocyte deformation and neuronal dysfunction (3). Among these disorders, chorea-acanthocytosis (ChAc) stands out as one of the principal autosomal recessive subtypes, highlighting the intersection of hematological abnormalities and neurological decline.

ChAc, caused by biallelic mutations in the VPS13A gene, is characterized by a deficiency of chorein, a protein essential for maintaining membrane integrity in erythrocytes and neurons (4). The ChAc mainly occurs in adulthood with an average age of about 35 years, and rarely occurs before the age of 20 years or after the age of 50 years (5). Clinically, ChAc was associated with a wide spectrum of phenotypes, typically including involuntary movements, cognitive decline, seizure, psychiatric features, and neuromuscular manifestations with elevated serum biochemical indicators and increased acanthocytes in peripheral blood (1, 6). Elevated liver enzymes were found in over 50% of patients. Acanthocytosis was observed in almost all ChAc patients, ranging from 5% to 50%, but didn't seem to correlate with the severity of ChAc (6). The common link between the neurological and erythroid abnormalities is an abnormality of cell membranes. Detection of acanthocytes in a blood film is diagnostically useful (7). Notably, the diagnosis of ChAc remains clinically challenging due to its phenotypic overlap with conditions such as Huntington's disease, McLeod syndrome, and Pantothenate kinase-associated neurodegeneration (PKAN).

To date, diverse mutation patterns of VPS13A, consisting of missense, nonsense, frameshift, splice site, replication, and deletion mutations, have been reported (8). In this report, we discovered two novel loss-of-function mutations, which have not been reported previously, and extended the genetic characteristics of ChAc. Detailed clinical features and Molecular Studies are presented. These findings expand the VPS13A variants spectrum and confirm the clinical variability in ChAc patients.

## 2 Methods

### 2.1 Patients and samples

Three patients were recruited from the Department of Neurology of the First Affiliated Hospital of Fujian Medical University, including DNA samples stored in the internal registry of the Department of Neurology and the Institute of Neurology and sent by clinicians for the diagnosis of extrapyramidal diseases during the years 2013–2022. Patients were examined by at least two senior neurologists, and their family members were also tested, if possible. Genomic DNA of patients and closest living relatives was extracted from peripheral lymphocytes using a TGuide Blood Genomic DNA Kit (Tiangen, Beijing). All samples were obtained with informed consent of probands or their legal representatives. The study was approved by the local ethics commission.

### 2.2 Whole exome sequencing

Whole-exome sequencing (WES) was performed for the two pedigrees. A SureSelect Human All Exon V6 kit (Agilent) was used to capture whole-exome DNA. Sequencing was performed by the Illumina HiSeq 3,000 platform and aligned to the consensus sequence (UCSC hg38). Genome Analysis Toolkit (GATK) and ANNOVAR were used to annotate the variants. Variants that met the following criteria were excluded first: (i) the variant did not affect a change in amino acid sequence; and (ii) the allele frequency was >1% in the 1,000 Genomes Project, ESP database, or gnomAD. Then we filtered the variants according to patterns of inheritance and obtained the potential disease-causing genes.

### 2.3 RNA splicing analysis

Total RNA was isolated from the peripheral blood leukocytes of family members. RNA was extracted using a Trizol extraction kit (Invitrogen, Carlsbad, CA, USA) and then synthesized to cDNA with a PrimeScript RT reagent kit (TAKARA BIO, Kusatsu, Shiga, Japan) according to the manufacturer's protocols. Primers were designed to target VPS13A exons 2-4 to confirm aberrant splicing (forward: 5′-GAGGAACATGGTTTTCGAGTC-3′; reverse: 5′-GTCCCGATTTGTGATATCATC-3′). The products amplified by the above primers were separated by agarose gel electrophoresis. The DNA fragments were then purified from the gels and sent for Sanger sequencing.

### 2.4 Sanger sequencing

Sanger sequencing was carried out to validate the potential variants identified by WES on an ABI 3,500 x L DxGenetic Analyzer (Applied Biosystems, Foster City, USA). Screen all family members with or without extrapyramidal symptoms.

### 2.5 Peripheral blood smear preparation

The morphology of RBCs was investigated using a method according to Alexander Storch et al. (9). Blood was drawn into 5 ml commercially available syringes pre-filled with potassium-EDTA solution (Changgeng, Fuzhou). Blood smears were prepared on glass slides and stained using the commercially available Wright-Giemsa Stain Kit (Baso, Zhuhai). All blood preparations were investigated on photomicrographs using a light microscope (DP27; Olympus, Japan) at a magnification of 1,000 (oil immersion). All RBCs on at least five photomicrographs of each preparation were analyzed by a blinded investigator. Corresponding to Redman's classification (10), type AI/AII RBC were counted as abnormal (referred here as acanthocytes). The number of acanthocytes was expressed as a percentage of total erythrocytes.

### 2.6 Scanning electron microscopy analysis

In our patients, fresh peripheral blood was screened for erythrocytes by environmental scanning electron microscopy (ESEM) according to previous studies (11). Briefly, aliquots of red blood cell suspensions were gently pelleted by centrifugation and fixed in 2.5% glutaraldehyde and 4% paraformaldehyde. After washing, the cells were dehydrated in a 50%−100% graded series of ethanol, dried by critical point, coated with a conducting material, and imaged with ESEM. A board-certified clinical pathologist independently determined the erythrocytes in a blinded manner. Contracted red cells with irregularly spaced thorny surface projections were defined as acanthocytes. The counts of acanthocytes within 3% were considered to be within the normal range (12).

## 3 Results

### 3.1 Identification of variants by whole exome sequencing and sanger sequencing

We identified 3 new ChAc cases from 2 families. Clinical features of all 3 cases were summarized in Table 1. WES was performed in 2 unrelated patients. After verification by Sanger sequencing, we found 3 variants within VPS13A (NM 033305) in two index cases, all of whom carried loss-of-function mutations. Among these novel variants without included in HGMD, one is homozygous splice-site mutation (c.145-2A > G), and the other two were compound heterozygous frameshift mutations (c.94_95insC), nonsense mutations (c.130A > T). The frequency of c.145-2A > G was absent in ExAC. The variants c.94_95insC and c.130A > T were absent in gnomAD. This mutation (c.145-2A > G) was predicted by MutationTaster as disease-causing. The other two variants (c.94_95insC; c.130A > T) were predicted by MutationTaster to be prediction disease causing. According to American College of Medical Genetics and Genomics (ACMG) standards, we classified these three variants as “likely pathogenic” (PVS1 and PM2).

**Table 1:** Summary of clinical features of the ChAc families.

**Family #**	**1**		**2**
Case #	II-2	II-1	II-1
Gene	VPS13A	VPS13A	VPS13A
Mutation	***c.145-2A** **>** **G***		***c.130A** **>** **T c.94_95insC***
Diagnosis	ChAc		ChAc
Age of onset	26	31	22
Gender	M	F	M
Initial Symptom	Involuntary movements	Asymptomatic hyperCKemia	Involuntary movements
Disease duration	2	3	10
Movement disorder	Orofaciolingual chorea, involuntary vocalizations, and Involuntary movements of the limbs	No	Orofaciolingual chorea, involuntary vocalizations, and alopecia
CK level	2091–2853 U/L	732 U/L	500–600 U/L
Visual impairment	No	No	No
Cognitive impairment	No	No	No
Cardiac disease	No	No	No
Peripheral neuropathy	Yes	No	No
Acanthocytosis	6.0%	NA	>10%

M, male; F, female; NA, not available.

### 3.2 RNA splicing analysis to enhance the pathogenicity of ACMG

**Figure 1:**
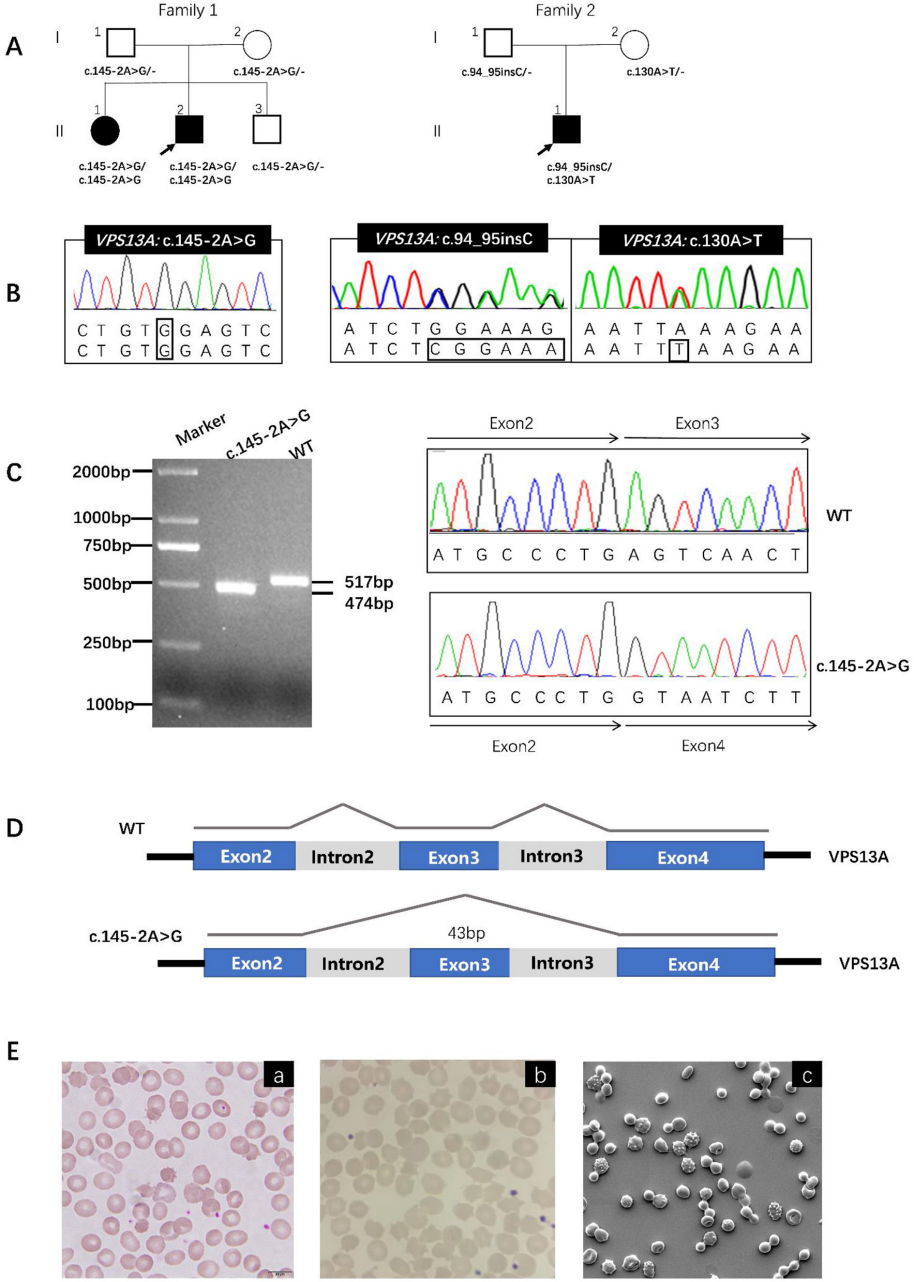
Pedigrees, mutation analysis, RNA Splicing Analysis, and peripheral blood smear for the ChAc families. **(A)** Pedigrees of three cases from two ChAc families: The square represents the male, and the circle represents the female. A filled symbol indicates the proband. **(B)** Mutation analysis: Visualization of the mutations in our cases is shown with Chromas. **(C)** RNA Splicing Analysis: Agarose gel electrophoresis analysis of VPS13A fragments in the proband (II-2, lane 2) and normal controls. The proband exhibits an aberrant 474 bp fragment (F1) compared to the wild-type 517 bp band, indicating exon skipping. Mutation visualization via Chromas confirms the splicing site mutation c.145-2A>G. **(D)** RNA Splicing Analysis: Schematic diagram illustrating the consequence of the c.145-2A>G mutation, which disrupts the canonical splice acceptor site of intron 2, resulting in complete skipping of exon 3 during transcript processing. **(E)** Peripheral blood smear: acanthocytes were shown via Wright-Gimsa staining (original magnification, ×400) in proband (II-2) in F1 (a), proband (II-1) in F2 (b). Examination of peripheral blood of proband (II-2) in F1 by scanning electron microscopy (original magnification, ×2500) showing acanthocytosis (c).

We further performed functional validation at the RNA level. Via detection by WES, ac.145-2A > G mutation in VPS13A in proband (II-2) in F1, causing ChAc, was identified. We therefore sought to validate the result through analysis of cDNA reverse transcribed from RNA extracted from the peripheral blood samples of the patient. A shorter transcript was observed in gel electrophoresis, indicating that the position of the lost acceptor for the variant led to exon 3 skipping, and the length of the absent exon (43bp) matched its observed length in the patient-derived cDNA sequence, compared with that in a healthy control. Sanger sequencing confirmed that exon 3 was absent in the truncated transcript due to transcriptional skipping caused by the c.145-2A > G variant (Figure 1).

### 3.3 Clinical features of patients carrying VPS13A variants

#### 3.3.1 Case 1

A 26-year-old male, who developed orofaciolingual chorea, involuntary vocalizations, and involuntary limb movements 2 years ago. Electromyography (EMG) testing demonstrated sensory conduction velocity slowing, abnormal spontaneous activities in the right first dorsal interosseous. Cognitive examination only showed reduced short-term memory (Mini Mental State Examination 28/30, MoCA 26/30). Laboratory testing revealed persistent hyperCKemia (2091–2853 U/L, normal 24–170 U/l), elevated serum CK-MB, LDH, and AST/ALT levels. His peripheral blood smear demonstrated 6.0% acanthocytes after Wright–Gimsa staining, and environmental scanning electron microscopy also observed RBC with spicules, which were irregular in shape and orientation/distribution (Figure 1). Nerve conduction studies (NCS) showed peripheral neurogenic alterations in the upper and lower limbs. The electrocardiogram presented QT prolongation (442 ms) while the echocardiogram was normal. His family is notable for an elder sister (aged 31; II-1 in F1) with hyperCKemia (732 U/L), elevated AST/ALT, LDH, and CKMB since her pregnancy 3 years ago, but with no chorea or dystonia.

#### 3.3.2 Case 1

**Figure 2:**
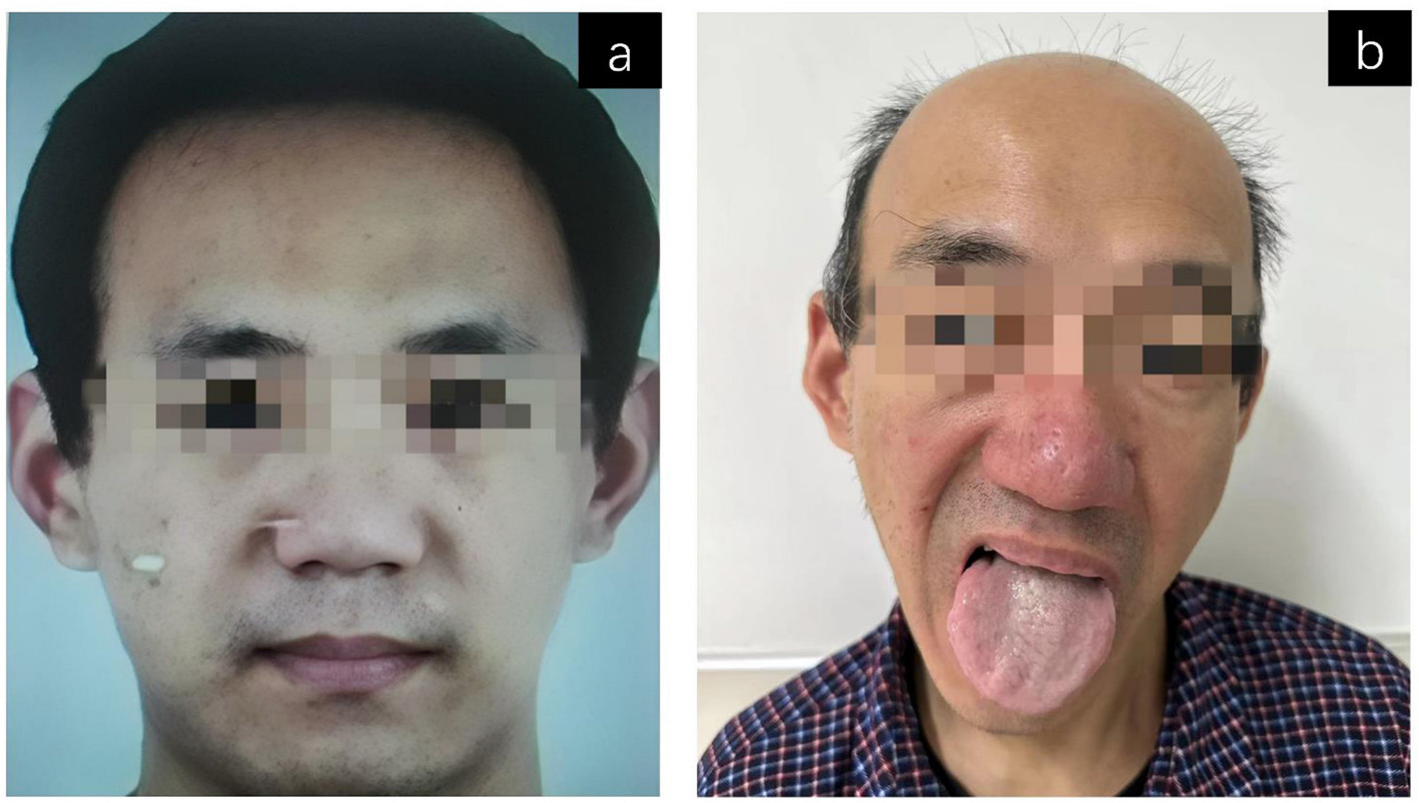
Clinical comparison of alopecia symptoms during disease progression. **(a)** Photos of the patient at the age of 30, showing uniform hair distribution, normal density, and no visible exposed areas of the scalp. **(b)** A photo of the patient at the age of 42. It has been 10 years since then. The picture shows obvious hair thinning and increased scalp visibility, tongue muscle tone disorder, and the tongue protruding to the right.

A 42-year-old male has suffered from chorea of the mouth and involuntary movements for the past 10 years. The patient shows increased tongue movement, with the tongue sticking out to the right. Occasionally, there are actions such as frowning, blinking, and sticking out the tongue. It often leads to biting the oral mucosa and causing oral ulcers. The gait characteristics of the patient are intermittent trunk flexion, extension spasm, and occasional knee flexion. The patient is prone to shaking their limbs when walking, and occasionally experiences head drooping and flexing of the right leg. The muscle strength of the limbs is normal, the muscle tone is reduced, and the tendon reflexes of the limbs are decreased. No Kaiser–Fleischer (K–F) rings were observed in the bilateral corneas. The cognitive function was hardly impaired. Laboratory tests showed persistent hyperemia (500–600 U/L). It is worth noting that the patient showed obvious hair loss during the course of the disease, which may be related to endocrine dysfunction secondary to underlying neurological diseases (Figure 2).

## 4 Discussion

Chorea-acanthocytosis (ChAc) is a rare neurodegenerative disorder, with an estimated global prevalence of 1 case per million population (1). While cases have been reported worldwide, higher incidence clusters are observed in regions with increased consanguinity rates, such as the Middle East and Japan (13). The disease typically manifests between the second and fourth decades of life, with a mean age of onset around 30 years, which aligns with the age range of our reported cases (14). ChAc is inherited in an autosomal recessive manner and caused by mutations in the VPS13A gene, which interfere with the production of chorein, found in brain tissue and erythrocytes (15). Recent studies have demonstrated that chorein and lipid scramblase XK are functional partners at the plasma membrane (16, 17). Chorein regulates many vital processes, including cytoskeletal architecture, calcium homeostasis, autophagy, and cell survival (18). Chorein binds to the cell membrane through the N-terminal PH domain, and the C-terminal β-helical structure is involved in protein-protein interactions. The destruction of functional domains can directly lead to neurodegeneration (19). Among the 73 exons of the VPS13A gene without specific hotspots, many disease-related mutations have been described. The total length of the gene is about 250 kb, which is composed of 73 exons (20). We reported three novel highly deleterious homozygous variants of the VPS13A gene, including a novel compound heterozygous VPS13A mutation (c.94_95insC and c.130A > T) and two novel homozygous mutations (both NM_033305.2:exon3:c.145-2A > G), which further expands the mutation spectrum of VPS13A.

The clinical manifestations observed in our cohort align with the core features of ChAc, including progressive chorea and elevated serum creatine kinase levels (21). Notably, one patient exhibited severe alopecia, a feature rarely documented in ChAc. While the pathophysiology of hair loss in this context remains unclear, we speculate that hair follicle dysfunction could stem from impaired lipid metabolism or cytoskeletal integrity in keratinocytes, as chorein is known to regulate membrane stability and vesicular trafficking in various cell types (22). Alternatively, chronic oxidative stress secondary to neurodegeneration or systemic metabolic disturbances in ChAc may disrupt hair follicle cycling (23). Previous studies have reported hypothalamic-pituitary axis abnormalities in ChAc patients (24). Further investigation, integrating trichoscopic analysis and lipidomic profiling of scalp tissues, could help clarify this association. Current therapeutic strategies for ChAc remain largely supportive and symptomatic (25). Chorea may be partially alleviated with dopamine-depleting agents or atypical antipsychotics, though their efficacy is variable and limited by side effects such as sedation or drug-induced parkinsonism (26). Neuropathic pain associated with peripheral neuropathy often requires gabapentin or duloxetine, while selective serotonin reuptake inhibitors (SSRIs) may address comorbid depression (27). Crucially, multidisciplinary care involving physiotherapy and speech therapy is essential to mitigate functional decline.

This report expands the mutational and phenotypic landscape of VPS13A-related ChAc, highlighting novel genetic variants and atypical clinical features such as alopecia. The integration of clinical observations with molecular insights underscores the systemic nature of chorein deficiency and calls for multidisciplinary investigations to unravel its pleiotropic effects. However, our study is limited by its small cohort size and the absence of functional validation for the identified variants, which precludes definitive conclusions about their mechanistic contributions to atypical features like alopecia. Future work should prioritize longitudinal studies in larger cohorts and experimental models to address these gaps and refine genotype-phenotype correlations in ChAc.

## 5 Conclusion

This study expounds the clinical characteristics and diagnostic approaches of chorea-acanthocytosis (ChAc), providing key insights for differentiating this neurodegenerative disease from other diseases with similar clinical symptoms. Although the detection of VPS13A gene mutations and the identification of chorein protein deficiency by Western blot remain the gold standard for diagnosis, the proposed multi-dimensional framework—integrating progressive motor disorders, neuropsychiatric manifestations, and hematological features—has significantly improved clinicians' ability to identify ChAc in overlapping phenotypes.

## Data Availability

The raw data supporting the conclusions of this article will be made available by the authors, without undue reservation.
